# Caregivers of patients with malignant pleural mesothelioma: who provides care, what care do they provide and what burden do they experience?

**DOI:** 10.1007/s11136-023-03410-4

**Published:** 2023-04-25

**Authors:** Adam Moore, Bryan Bennett, Gavin Taylor-Stokes, Melinda J. Daumont

**Affiliations:** 1 Adelphi Real World, Bollington, UK; 2 Bristol Myers Squibb, Uxbridge, UK; 3 Bristol Myers Squibb, Braine-L’Alleud, Belgium

**Keywords:** Mesothelioma, Malignant, Quality of life, Patient care, Caregiver burden

## Abstract

**Purpose:**

There are limited data on the impact of caregiving for patients with malignant pleural mesothelioma (MPM) on the caregiver. We aimed to identify the demographic characteristics of these caregivers, the caregiving activities they perform and how caregiving burden impacts their work productivity and overall activity.

**Methods:**

This cross-sectional study collected data from caregivers of patients with MPM across France, Italy, Spain and the United Kingdom January-June 2019. Caregiver demographics, daily caregiving tasks and the impact of caregiving on physical health was collected via questionnaire. The Zarit Burden Interview (ZBI) was used to assess caregiver burden and the Work Productivity and Activity Impairment questionnaire (WPAI) assessed impairment at work and during daily activities. Analyses were descriptive.

**Results:**

Overall, 291 caregivers provided data. Caregivers were mostly female (83%), living with the patient (82%) and their partner/spouse (71%). Caregivers provided over five hours of daily emotional/physical support to patients. ZBI scores indicated 74% of caregivers were at risk of developing depression. Employed caregivers had missed 12% of work in the past seven days, with considerable presenteeism (25%) and overall work impairment (33%) observed. Overall, the mean activity impairment was 40%.

**Conclusion:**

Caregivers provide essential care for those with MPM. We show caregiving for patients with MPM involves a range of burdensome tasks that impact caregivers’ emotional health and work reflected in ZBI and WPAI scores. Innovations in the management of MPM must account for how caregivers may be impacted and can be supported to carry out this important role.

**Supplementary Information:**

The online version contains supplementary material available at 10.1007/s11136-023-03410-4.

## Introduction

Malignant pleural mesothelioma (MPM) is a tumour that develops in the mesothelial surfaces of the pleura. It is associated with exposure to asbestos, with asbestos workers and those residing near asbestos manufacturing plants being at increased risk of developing MPM [1–4]. MPM has a long latency period of around 40 years, patients are often older males with a mean age of approximately 70 years at diagnosis, a median survival time of 8–14 months from diagnosis, and a 5-year survival rate of 10% [5–9].

MPM is relatively rare with 34,614 new cases reported globally in 2017. Until recently, cases had been rising across most countries; it was only 20–30 years after banning asbestos use that countries like France, Italy, Spain, and the United Kingdom (UK) started to see a decline in cases [10, 11]. Given the long latency, cases are likely to continue to rise in countries slower to enact a complete ban as many countries are yet to reach expected peak death rates [12–14].

MPM is highly aggressive [15], with a majority of patients presenting with late-stage unresectable disease that responds poorly to treatment [16, 17]. Previously there was little innovation in successful treatment for over a decade and treatment options remained limited for patients with MPM. More recently, nivolumab and ipilimumab immunotherapy has been approved in the United States (US) [18] and is recommended in the European Society for Medical Oncology guidelines [19]. Patients and their caregivers will experience prolonged periods of treatment (many treatment cycles followed by maintenance therapy) and the burden associated with treatment.

MPM is associated with an overall sense of physical debilitation and poor health, depression, anxiety, and cognitive impairment [20]. Given the highly symptomatic and aggressive nature of MPM, many patients require the assistance of caregivers [21]. Caregivers are often spouses, female and over 50 years of age [20, 22]. Due to the rapid progression of MPM and its physical and emotional toll, caregiver burden is likely to be high. However, there are limited data on caregiver burden in MPM and thus it has remained poorly understood.

Caregiver burden has been defined by a multidimensional approach, considering the physical, emotional, psychological, social and financial impacts on caregivers [23, 24]. The limited data on caregiving in MPM has shown that emotional functioning of caregivers is significantly impaired, with caregivers reporting higher levels of personal distress, receiving less support, and feeling less well-informed throughout the process of diagnosis than the patients they care for [20]. In addition, caregivers have poorer physical health than healthy peers and higher rates of depression and intrusive thoughts about death than the patients they care for [25].

In many countries, informal caregiving represents the backbone of the social care delivery system [26], providing an estimated annual economic value of €576 billion in 2016/2017 [27]. Informal caregivers are estimated to make up 10–25% of the total population of caregivers in Europe [28] and provide 80% of long-term care [29]. Therefore, without informal caregivers, societal costs, including health and social care costs would likely be severe. Thus, it is important to understand the burden placed on informal caregivers to ensure they receive the required support. Until recently, support for MPM was based on existing care infrastructures established for lung cancer patients; however, this fails to recognise the different needs in MPM [22]. One study found patients with MPM felt more hopelessness than patients with lung cancer—perhaps owing to fewer successful treatment options in MPM—and many felt anger that their disease resulted from bad workplace practices [30]. Further distress has been linked to delays in MPM diagnoses and uncertain prognoses [22]. These studies highlight how the challenges faced by patients with MPM and their caregivers may differ from patients with lung cancer.

There is a paucity of research on MPM caregiving, with much of the information about caregiver burden being anecdotal [20]. Therefore, there are many gaps in our understanding of caregiver burden in MPM. We aimed to identify the demographic characteristics of caregivers of patients with MPM, the caregiving activities they perform, and which of these were the most troublesome. We also investigated the burden of caregiving, and its impact on the caregivers’ health, work productivity and overall activity. We describe these data stratified by the clinical characteristics of the patients with MPM being cared for to further understand how these characteristics impact the caregiver’s role and associated burden.

## Methods

### Study design

Data were drawn from a larger multinational survey of physicians, patients with MPM and their caregivers in a real-world setting that included retrospective and cross-sectional data collection [31]. Physicians abstracted retrospective data from their next 5–10 eligible consulting patients’ medical charts. Each patient and any primary caregiver accompanying the patient to a consultation were invited to complete a patient or caregiver questionnaire. Physicians were selected by local data collection agencies. Physicians had to be specialists in oncology or pulmonology, qualified for 5–35 years, personally responsible for the management of patients with unresectable MPM and have consulted with at least five patients with MPM over the past three months. Patients were adults (aged over 18 years), had a physician-confirmed diagnosis of unresectable MPM and were not participating in a clinical trial. Data from patients and physicians on treatment patterns and patient burden have previously been published [31].

### Participants

In this report, we focused on caregiver survey data. Caregiver data were collected from 291 caregivers across France, Italy, Spain and the UK between January and June 2019, allowing us to collect caregiver perspectives across a variety of healthcare systems in Europe.

Caregivers eligible for inclusion in the current study were aged 18 years or over and the primary caregiver (spouse, partner, child, other relative or friend) providing informal (unpaid) care for a patient diagnosed with unresectable MPM.

In total, there were 297 patients from the overall survey that had a caregiver recorded within their medical record but no corresponding caregiver self-completion questionnaire (CSC). Whilst exact response rates were not captured, our sample of 291 caregivers suggests a response rate of close to 50%.

### Study measures

Each caregiver filled out a CSC. Demographic information was collected including age, sex, living situation (with or not with patient), relationship to patient, employment status and household income.

To identify caregiving tasks undertaken daily, caregivers were given a list of 22 options of daily tasks and asked to select as many as were applicable to them and their top three most troublesome activities.

To measure overall impact on health, caregivers were asked to rate the impact caregiving had on their health with response choices on a 7-point numerical rating scale from 1 = “did not impact my health at all” to 7 = “severely impacted my health”.

The Zarit Burden interview (ZBI) assessed the level of caregiver burden [32] and has been shown to be a reliable and valid measure of caregiver burden [33, 34].Validated translations were used. The ZBI is a 22-item questionnaire designed to measure subjective degree of burden on a 5-point scale, for each question caregivers selected from 0 = “never” to 4 = ”almost always”. The ZBI comprises questions that fit into different domains, the scores for each domain are calculated by adding the cumulative scores given for each question in that domain. The domains are burden in the relationship (range = 0–24), emotional well-being (range = 0–28), social and family life (range = 0–16), loss of control over one’s life (range = 0–16), finances (range = 0–4), personal strain (range = 0–48) and role strain (range = 0–24). ZBI total score is the sum of scores and ranges between 0 and 88, with higher scores indicating greater burden [35]. Schreiner et al. identified a statistically valid threshold score of 24 for the ZBI to identify caregivers at risk for depression, whereby caregivers with this score or higher were considered at risk of developing depression [36].

The Work Productivity and Activity Impairment questionnaire (WPAI) was used to assess caregivers’ impairment at work during the past seven days. WPAI is a validated measure that consists of four scores: absenteeism (work time missed), presenteeism (impairment at work/reduced on-the-job effectiveness), work productivity loss (overall work impairment) and activity impairment (regular activities other than work) [37]. WPAI scores range from 0–100 (expressed as impairment percentages), with higher scores indicating greater impairment. The versions administered had undergone independent translations, harmonization, back-translation, expert review, and review by local language users [38].

### Data analysis

All analyses were conducted using Stata v16 [39]. Caregivers were stratified by country, age, patients’ current line of treatment, ECOG performance score (Eastern Cooperative Oncology Group—higher scores indicate greater difficulty caring for themselves) of the patient and patient MPM subtype.

No formal hypotheses were developed prior to conducting this study and all analyses were descriptive in nature with no statistical comparisons conducted. Missing data were not imputed. The number of observations is reported for each variable. In general, missingness was very low, with no missing data observed for variables related to caregiving activities or the ZBI. Continuous variables were described as means and standard deviations (SD) and categorical variables were described as numbers and percentages.

## Results

### Demographics

Overall, 291 CSC forms were collected for caregivers of patients with MPM from France (n = 90), Italy (n = 70), Spain (n = 111) and the UK (n = 20). Patient demographics for whom the caregivers in this study provided care are presented in Table 1. The table also includes demographics of patients included in the medical chart abstraction that had a known caregiver that did not complete a CSC (n = 297). The effect size of differences between these cohorts was ≤ 0.2 for all demographic and clinical characteristics.

**Table 1:** Demographics and clinical characteristics of the patients for whom care was being provided

Patient demographics	Patient + caregiver + CSC (n = 291)	Patient + caregiver + no CSC (n = 297)
Country, n (%)		
France	90 (31)	66 (22)
Italy	70 (24)	97 (33)
Spain	111 (38)	56 (19)
United Kingdom	20 (7%)	58 (20)
Age, years (SD)		
Mean	67.2 (8.3)	69.1 (9.1)
Sex, n (%)		
Female	56 (19)	59 (20)
Smoking status, n (%)		
Current	59 (20)	60 (20)
Ex-smoker	158 (54)	153 (52)
Never smoked	71 (24)	80 (27)
Not stated	3 (1)	4 (1)
Employment status, n (%)		
Full-time	5 (2)	13 (4)
Part-time	7 (2)	13 (4)
On long-term sick leave	61 (21)	34 (11)
Home maker	14 (5)	15 (5)
Student	0 (0)	0 (0)
Retired	198 (68)	218 (73)
Unemployed	4 (1)	4 (1)
Not stated	2 (1)	0 (0)
Clinical characteristics		
Current line of treatment, n (%)		
1L SACT	174 (60)	162 (55)
1L maintenance	35 (12)	27 (9)
2L + SACT	34 (12)	27 (9)
2L + BSC	48 (16)	81 (27)
Resection status at diagnosis, n (%)		
Resectable	17 (6)	18 (6)
Unresectable	271 (93)	273 (92)
Resection status not known	3 (1)	6 (2)
MPM stage at diagnosis, n (%)		
Stage 1	9 (3)	14 (5)
Stage 2	21 (7)	19 (6)
Stage 3	67 (23)	74 (25)
Stage 4	192 (66)	183 (62)
Unable to stage	0 (0)	3 (1)
Unknown	1 (< 1)	4 (1)
ECOG performance score at diagnosis, n (%)		
0	48 (16)	53 (18)
1	158 (54)	169 (57)
2 +	82 (28)	69 (24)
Unknown/missing	3 (1)	6 (2)
Histology (MPM subtype), n (%)		
Epithelioid	186 (64)	198 (67)
Biphasic	65 (22)	59 (20)
Sarcomatoid	29 (10)	32 (11)
Unknown	11 (4)	8 (3)

Data for patients that were receiving caregiver support (as noted in their medical record) but had no corresponding CSC were included for comparison (n = 297)

Best supportive care, *BSC*; caregiver self-completion form received, *CSC*; Eastern cooperative oncology group, *ECOG*—higher scores indicate lower performance status; first-line,*1L*; malignant pleural mesothelioma, *MPM*; systemic anti-cancer therapy, *SACT*; second-line, 2L; *SD*, standard deviation

Caregivers had a mean age of 59 years, 83% were female and 82% were living with the patient they cared for, with 71% being the patient’s spouse/ partner (caregivers that were spouses/ partners had a mean age of 63 years). In total, 36% of caregivers were working alongside their caregiving duties and 38% were retired (Table 2).

**Table 2:** Demographics of caregivers that provided a caregiver self-completion form

	All caregivers (n = 291)
Caregiver demographics	
Country, n	n = 291
France	90
Italy	70
Spain	111
UK	20
Age, years (SD)	
Mean	59.0 (12.0)
Sex, n (%)	
Female	241 (83)
Living situation, n (%)	
Lives with the patient	240 (82)
Does not live with the patient	50 (17)
Unknown	1 (< 1)
Relationship to patient, n (%)	
Partner/spouse	207 (71)
Parent	0 (0)
Friend/neighbour	11 (4)
Child	46 (16)
Sibling	13 (4)
Other	4 (1)
Other family member	5 (2)
Unknown	5 (2)
Employment status, n (%)	
Working full time	67 (23)
Working part time	39 (13)
Student	0 (0)
Homemaker	55 (19)
Retired	110 (38)
Unemployed	14 (5)
Other	4 (1)
Unknown	2 (1)
Household income (UK), n (%)	n = 20
Less than £10,000	1 (5)
£10,001–£20,000	2 (10)
£20,001–£30,000	3 (15)
£30,001–£40,000	1 (5)
£40,001–£50,000	3 (15)
£50,001–£60,000	3 (15)
£60,001–£70,000	0 (0)
£70,001–£80,000	2 (10)
£80,001–£90,000	0 (0)
More than £90,000	0 (0)
I prefer not to answer	5 (25)
Unknown	0 (0)
Household income (EU), n (%)	n = 271
Less than €10,000	9 (3)
€10,001–€20,000	33 (12)
€20,001–€30,000	64 (24)
€30,001–€40,000	43 (16)
€40,001–€50,000	14 (5)
€50,001–€60,000	8 (3)
€60,001–€70,000	1 (< 1)
€70,001–€80,000	1 (< 1)
€80,001–€90,000	0 (0)
More than €90,000	3 (1)
I prefer not to answer	86 (32)
Unknown	9 (3)

European Union, *EU*; standard deviation, *SD*; United Kingdom, *UK*

### Caregiving activities

**Fig. 1:**
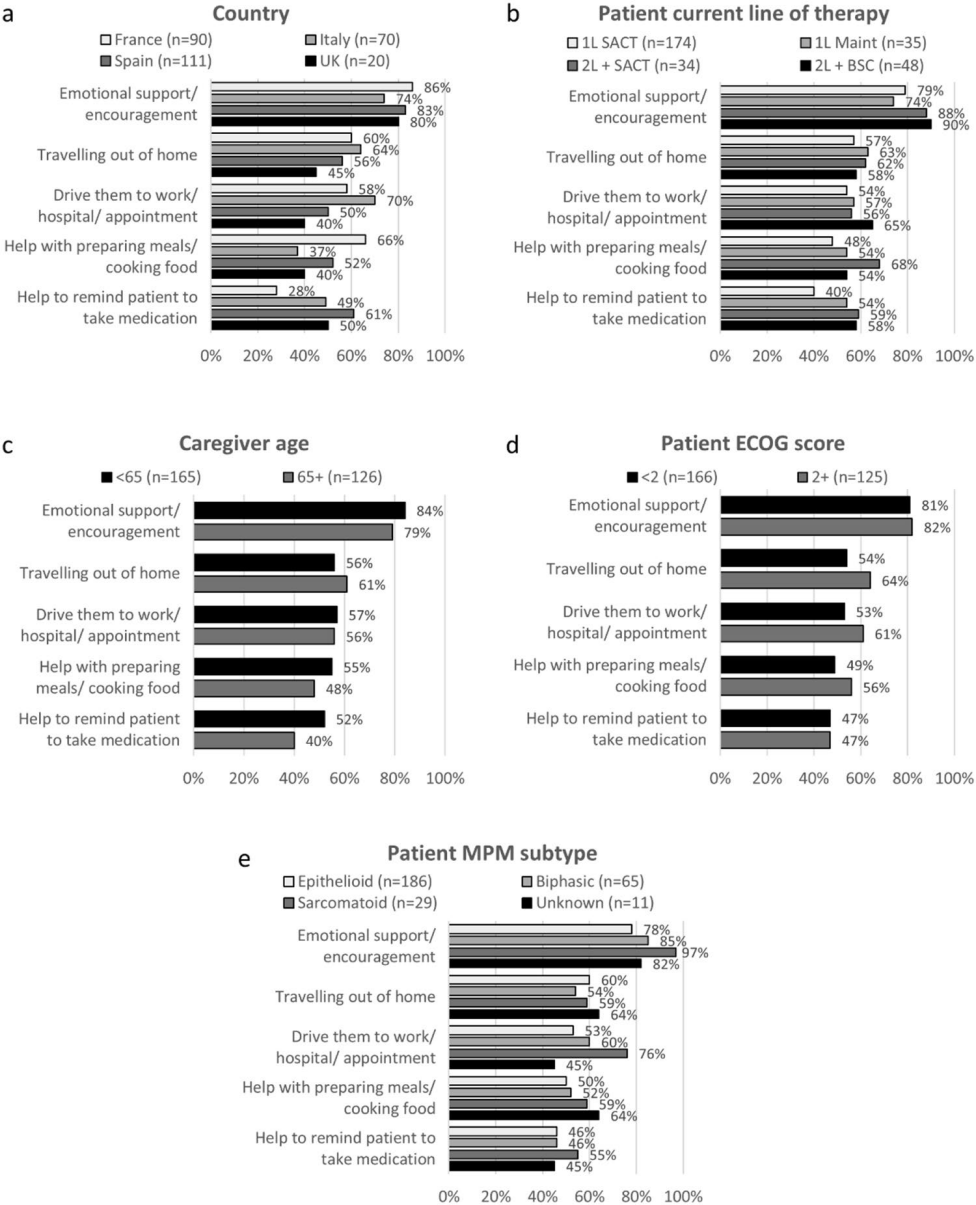
Top five caregiving activities undertaken daily by caregivers of patients with MPM (% of caregivers completing each task daily). Data are stratified by country, patient current line of treatment, caregiver age, ECOG score and MPM subtype. Best supportive care, *BSC*; caregiver self-completion form received, *CSC*; Eastern cooperative oncology group, *ECOG*—higher scores indicate lower performance status; first-line,*1L*; maintenance, maint; malignant pleural mesothelioma, *MPM*; systemic anti-cancer therapy, *SACT*; second-line, *2L*; United Kingdom, *UK*

Caregivers spent an average of 5.8 (SD: 6.3) hours of their day providing emotional or physical support; the most frequently reported caregiver task was providing emotional support and encouragement, with 81% of caregivers reporting that the patient required this support daily. The next most common caregiving activities reported were travelling out of home (58%), driving patients to work/ hospital/ appointments (56%) and helping with preparing meals (52%). Figure 1 presents the stratified caregiving activities data. The proportion engaging in these activities daily was generally higher for caregivers of patients receiving second-line (2L) + systemic anti-cancer therapy (SACT) and 2L + best supportive care (BSC), than patients on first-line (1L) SACT or 1L maintenance, those caring for patients with the sarcomatoid subtype of MPM and when caring for patients with ECOG 2 and above.

Overall, the top five most troublesome caregiving activities reported were: providing patients with emotional support/encouragement (49%), driving the patient to work/hospital/appointments (28%), helping the patient travel out of their home (20%), getting patient dressed/washed (18%) and help with preparing meals (13%).

**Fig. 2:**
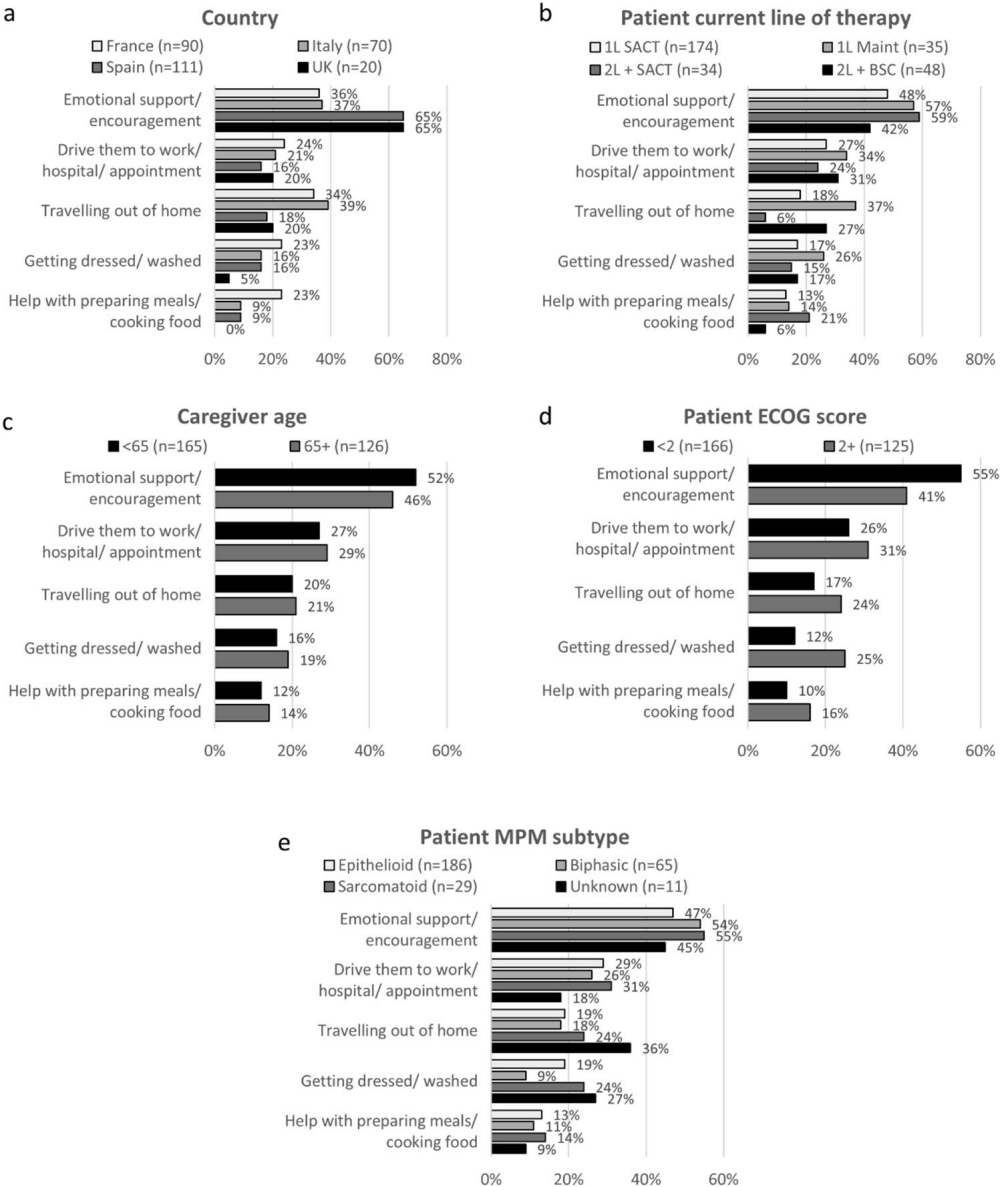
Top five most troublesome caregiving activities undertaken daily by caregivers of patients with MPM (% of caregivers selecting each task within their top three most troublesome tasks). Data are stratified by country, patient current line of treatment, caregiver age, ECOG score and MPM subtype. Best supportive care, *BSC*; caregiver self-completion form received, *CSC*; Eastern cooperative oncology group, *ECOG*—higher scores indicate lower performance status; first-line,*1L*; maintenance, maint; malignant pleural mesothelioma, *MPM*; systemic anti-cancer therapy, *SACT*; second-line, *2L*; United Kingdom, *UK*

We found some differences across countries in the perceived burden of different activities, with one in three caregivers in France and Italy reporting that providing emotional support was one of the top three most troublesome tasks compared with two in three caregivers in Spain and the UK (Fig. 2).

### Caregiver burden (ZBI)

**Fig. 3:**
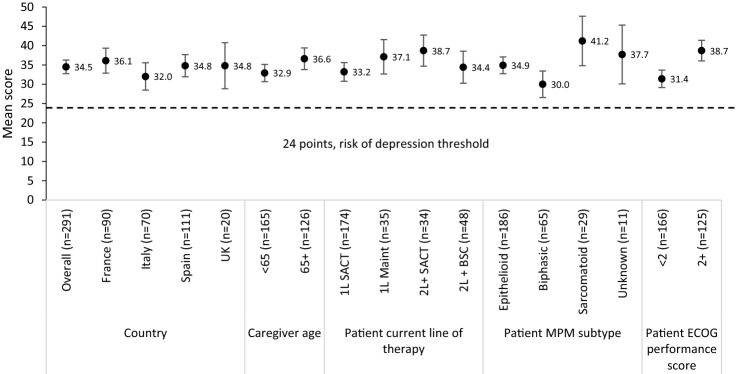
Mean ZBI-total stratified by country, age, patients’ current treatment, current line of treatment, disease subtype and ECOG performance score. Error bars represent 95% confidence intervals. Best supportive care, *BSC*; caregiver self-completion form received, *CSC*; Eastern cooperative oncology group, *ECOG*—higher scores indicate lower performance status; first-line,*1L*; maintenance, maint; malignant pleural mesothelioma, *MPM*; systemic anti-cancer therapy, *SACT*; second-line, *2L*; United Kingdom, *UK*; Zarit burden interview scale, *ZBI*

Caregivers’ overall mean (SD) ZBI total score was 34.5 (15.3). Overall, 74% of caregivers had a ZBI score of ≥ 24 (indicating high risk of depression) and the mean total score of all subgroups was ≥ 24. Caregivers of patients with worse performance score (ECOG > 2) or with sarcomatoid MPM reported experiencing higher burden (Fig. 3). Similarly, caregivers of patients at 1L-maintenance and 2L + experienced higher burden than those caring for patients at 1L-SACT.

Caregivers of older patients (65 + years) and those with poor performance status (ECOG > 2) reported higher ZBI scores across all domains than caregivers of younger patients (< 65 years) or with better performance status (ECOG < 2). Caregivers of patients with the sarcomatoid subtype of MPM reported higher burden in all domains compared with those caring for patients with other MPM subtypes. Caregivers of patients currently receiving 2L + BSC had lower burden than caregivers of patients currently receiving 1L-maintenance and 2L + SACT treatment (see Supplementary Fig. 1a–g). Although descriptive differences in ZBI total scores were observed between subgroups, these differences were cumulative across subdomains rather than being due to differences in individual domains.

### Impact of caregiving on physical health

**Fig. 4:**
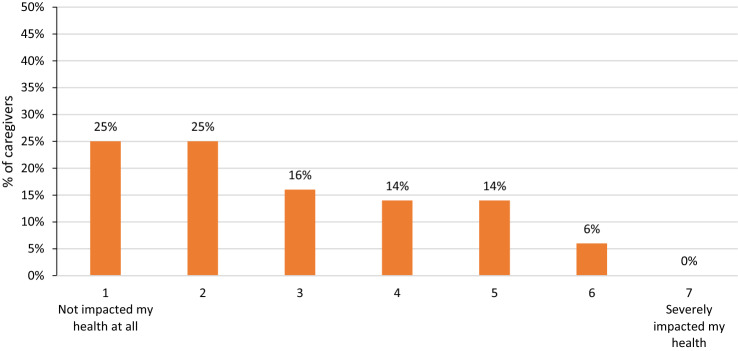
Caregivers (n = 291) self-reported impact of caregiving on health

In total, 75% of caregivers reported that caregiving had impacted their health (Fig. 4). Overall, 63% of caregivers were taking medication to treat a condition that they believed had been brought on or exacerbated due to caregiving.

### Impact of MPM on caregiver work and activity

**Fig. 5:**
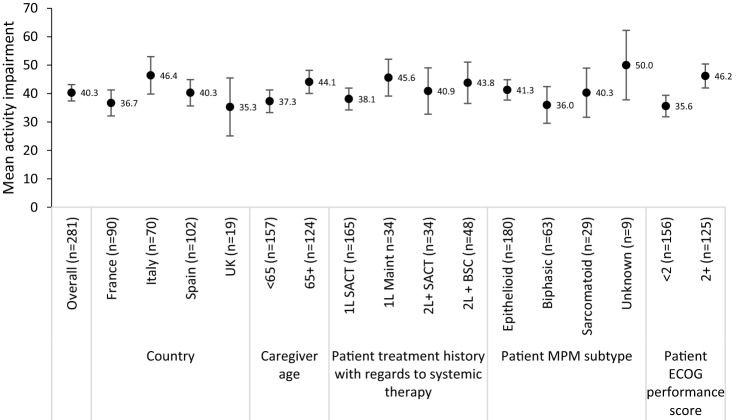
Mean overall activity impairment (WPAI) in caregivers of patients with MPM, stratified by country, age, patients’ current line of treatment, disease subtype and ECOG performance score. Error bars represent 95% confidence intervals. Best supportive care, *BSC*; caregiver self-completion form received, *CSC*; Eastern cooperative oncology group, *ECOG*—higher scores indicate lower performance status; first-line,*1L*; maintenance, maint; malignant pleural mesothelioma, *MPM*; systemic anti-cancer therapy, *SACT*; second-line, *2L*; United Kingdom, *UK*; work productivity and activity impairment questionnaire, *WPAI*

During the seven days prior to data collection, caregivers reported missing approximately 12% of work time, were impaired while working 25% of the time and their overall work impairment was 33%. Overall, the mean degree of activity impairment for caregivers of patients with MPM was 40% (Table 3). Activity impairment was highest for caregivers of patients > 65 years old, caregivers of patients currently receiving 1L maintenance and caregivers of patients with ECOG performance scores > 2 (Fig. 5).

**Table 3:** Overall work productivity among caregivers

	n	Mean (SD)	95% CI upper	95% CI lower
WPAI: percentage work time missed due to problem^†^	79	12.1 (17.8)	16.0	8.2
WPAI: percentage impairment while working due to caregiving^†^	91	24.9 (20.5)	29.1	20.7
WPAI: percentage overall work impairment due to caregiving^†^	78	33.3 (25.2)	27.7	38.9
WPAI: degree of activity impairment (expressed as impairment %)	281	40.3 (24.6)	37.4	43.2

All 291 caregivers were eligible to complete the activity impairment item

Confidence interval, *CI*; standard deviation, *SD*; work productivity and activity impairment questionnaire, *WPAI*

^†^Only caregivers that were currently employed (n = 106) were eligible to complete the WPAI work-related items

## Discussion

This study included data from a sample of caregivers across four European countries. The results provide a greater understanding of who is providing care to patients with MPM, the specific tasks undertaken daily and which tasks caregivers found most troublesome. It also explores caregiver burden and impact on physical and emotional health and impairment of caregiver work and activity.

An MPM diagnosis may require changes in daily activities and work and may result in changed roles within the family unit [40]. Most caregivers in this study lived with and were a family member of the patient (9 in 10) and 7 in 10 were their spouse/partner. This study also found caregivers of patients with MPM engaged in a wide range of daily activities including, supporting patients travelling out of the home, driving to work/hospital/appointments and helping with meals and medication management. Other studies have also shown that caregivers who are family members may be expected to spend a lot of time providing practical support involving such tasks [41].

In addition to various practical tasks, most caregivers in this study reported that they were providing daily emotional support and encouragement to the patient, and this activity was cited as being one of the top three most troublesome caregiving activities. Our findings were similar to a study of caregiving in lung cancer patients where a third of caregivers reported managing patients’ emotions as being one of the most challenging tasks they perform [42], and similar findings have been observed among caregivers of patients with other cancers [43]. The results also indicated potential cultural differences may impact the perception of burden, for example, we found one in three caregivers in France and Italy reported providing emotional support was one of the top three most troublesome tasks compared with two in three in Spain and the UK. This suggests cultural differences may impact perceptions of the burden of specific caregiving tasks. Variations in compensation available to MPM patients across countries may also play a role. For example, comprehensive compensation is available in France and the UK at 4.60 and 1.03 times the median income per year, respectively, [44] but in Spain, many cases may be under-recognised under existing compensation systems [45].

A cancer diagnosis, as well as the subsequent phases of the disease and its treatment, can be a source of intense stress both for the patient and the family as they face the challenge of uncertainty, treatment routines, and the threat of treatment failure [46]. Poor prognosis for patients with MPM and lack of treatment options may cause ever-increasing emotional challenges for the patient; many MPM patients and caregivers indicate their psychosocial care needs are not being met [47]. Harrison et al. showed caregivers receiving a referral to a specialist palliative care team were significantly more satisfied than those that did not, particularly because of the emotional support provided by these services [48]. Further, many patients may experience resentment and blame if their cancer is attributable to their work environment [30]. These factors are likely to cause disappointment and stress for the caregiver [20] as well as requiring them to support the patient through these challenges, likely resulting in stress, negative emotions, and role strain, which contributes to physical and psychological health impairment [46]. Warby et al. reported even in cases where caregivers were satisfied with treatment, they reported needing more communication about available treatments, end-of-life assistance and support after the patient’s death [49].

The level of perceived burden experienced by caregivers of patients with MPM observed in this study was higher than the burden reported for caregivers of advanced non-small cell lung cancer patients [50] and advanced cancer patients in a palliative day care setting [51], but similar to the levels of burden observed for caregivers of cancer patients within six months of diagnosis [52]. Our finding that poor ECOG performance score was a key driver of caregiver burden in MPM is consistent with findings in other cancers [50, 53]. Of note, our study found that caregivers of patients receiving BSC had lower burden than caregivers of patients receiving 1L maintenance and 2L + SACT. These patients may be receiving more external support in a palliative setting which could lead to lower burden on the informal caregiver. Further research should seek to identify optimal times when additional support may be introduced to support both the patient and caregiver to alleviate burden.

An earlier study reported patients with MPM receiving maintenance therapy had better health states and quality of life (QoL) than patients receiving 1L SACT or 2L + SACT [31]. However, we found that the caregivers of patients receiving 1L maintenance were experiencing similar burden to caregivers of patients that had progressed to 2L SACT. This result may reflect patients experiencing ongoing treatment cycles and visits for treatment/ management. This will not only have a psychological effect on the patient and caregiver but also involve more time spent travelling out of the home and driving patients to hospital, both tasks caregivers reported as being among the most troublesome.

Across all subgroups, the levels of burden observed for caregivers of patients with MPM in this study exceeded the threshold for risk of depression identified by Schreiner et al. [36]. Caregivers are at risk for several mental health problems including depression, anxiety, hopelessness, and social isolation [54, 55]. Evidence regarding the potential psychological costs of caregiving and its relationship to caregiver burden has been growing [56]. A recent study found that subjective caregiver burden, as measured by the ZBI, was associated with poorer physical and mental health for caregivers [57]. Studies using other measures of caregiver burden have found a similar positive relationship between increased burden and increased risk of developing anxiety and depression [58]. Emotional concerns were also one of the most identified problems for caregivers in a literature review on the effects of caring for a patient with cancer [59] and declines in psychological wellbeing have been reported for caregivers of lung cancer patients [60, 61]. Caregiver burden is frequently overlooked by physicians and the findings of this study further demonstrate the need for caregiver assessment and intervention [54].

Despite caregivers being essential to the healthcare system, the health of caregivers is not being prioritized. Many caregivers have been described as “hidden patients” [62], susceptible to a variety of stress-related illnesses (including depression) that often go untreated because there is no one to attend to the caregivers’ responsibilities while they recover [63]. Without providing adequate resources in long-term care services, demands on caregivers’ attention and time are likely increased, putting them at risk of developing serious mental and physical health problems. Only a quarter of the caregivers in this study reported that caregiving had not impacted their health. It is important that caregivers are supported in taking care of their own health needs as well as those of the patient they care for. However, for some, this will require them to overcome a culture that encourages caregivers to meet the needs of the patient at the expense of their own [64]. Where systems have been found to be inadequate or not supportive enough of caregivers, levels of anxiety and depression have remained high [65]. Caregivers that have received psychological and social resources reported lower levels of depression than those that have not [66]. In particular, support targeted at specific caregivers’ needs is often the most helpful [67].

In addition to our study providing insights into the burden of caregiving in MPM and the potential impacts to emotional and physical health, we also found that caregivers spent 40% of their daily activities impaired and those that were still working had 12% absenteeism and 25% presenteeism during the past seven days. Family and medical leave policy reform is needed to support caregivers. For example, in the UK, an employer is not legally obligated to pay a caregiver that takes time off to provide care, likely leading to caregivers returning to work before they are ready. There is growing interest from insurers and healthcare systems to understand caregiver burden both for caregivers themselves, the patients and the impact of caregiving on society. Studies such as this are vital across diseases if the required support is to be identified and provided to caregivers and further incorporated into insurer and healthcare system decision making. Importantly, caregiver burden has been found to be associated with factors linked to disease progression such as the ECOG performance score of patients [50] and patient QoL [64], with burden for caregivers increasing as ECOG performance score declines or QoL deteriorates. Recently, the European Medicines Agency and US Food and Drug Administration have approved nivolumab and ipilimumab combination therapy in the 1L setting [68, 69] based on the results of a clinical trial which demonstrated an increased time to QoL deterioration and prolonged survival [70–73]. Given the relationship between clinical outcomes and caregiver burden, improvements in treatment options for patients with MPM may be expected to lead to improved outcomes for caregivers also. Payers should consider caregiver burden when economically evaluating potential new treatments.

## Study strengths/limitations

A key strength of this study was the geographical spread of the primary caregivers, which provided a diverse and sizable caregiver population for evaluating the impact of this rare cancer on caregivers of patients in routine clinical care in Europe. As with all point-in-time study designs, the current study provides a snapshot of caregiver status only; therefore, the status of caregivers prior to taking on their caregiving role is unknown.

A limitation of this study was that it relied on the accuracy of recall by caregivers, although the selection of validated instruments that require short recall time was expected to minimise these effects. Caregiver inclusion was based on their willingness to participate, which has an inherent risk of selection bias. However, the demography of the patients with caregivers that did not provide a CSC was similar to that of those who did provide a CSC. Finally, as this study was purely descriptive, differences observed may not be statistically meaningful.

## Conclusion

The results of this multi-country, real-world study provide evidence that caring for patients with MPM involves a range of burdensome activities impacting caregivers’ emotional and physical health and their ability to undertake daily activities. Future innovation in the management of MPM will need to ensure improved outcomes for patients are not at the detriment of caregivers, and it is crucial caregivers receive the necessary support to carry out this important role. Interventions that maintain patient ECOG performance score and QoL by delaying disease progression are expected to improve caregiver outcomes. Focussing on maintaining caregiver health alongside the health of patients with MPM will also improve outcomes for caregivers and thus their ability to provide essential care for those with MPM.

## Supplementary Information

Below is the link to the electronic supplementary material.

Supplementary file1 (DOCX 43 KB)

Supplementary file2 (DOC 87 KB)

## Data Availability

All data relevant to the study are included in the article or uploaded as supplementary information. BMS policy on data sharing may be found at https://www.bms.com/researchers-and-partners/independent-research/data-sharingrequest-process.html.
