# Supplementary material for: Caregivers of patients with malignant pleural mesothelioma: who provides care, what care do they provide and what burden do they experience?
Source: Qual Life Res. 2023 Apr 25;32(9):2587–99. doi: 10.1007/s11136-023-03410-4 (PMC10393857; doi:10.1007/s11136-023-03410-4)
Supplement: Supplementary file 1 — Supplementary file1 (DOCX 43 KB) [file 11136_2023_3410_MOESM1_ESM.docx]

**Supplementary figures**

Supplementary figure 1a-g. Mean scores of caregivers across all ZBI domains stratified by country, age, patients’ current line of treatment, MPM subtype and ECOG performance score

a

b

c

d

e

f

g

Error bars represent 95% confidence intervals. Higher ZBI scores indicate greater burden.

Abbreviations: Best supportive care, BSC; Eastern Cooperative Oncology Group, ECOG – higher scores indicate lower performance status; first-line, 1L; maintenance, maint; malignant pleural mesothelioma, MPM; SACT, systemic anti-cancer therapy; second-line, 2L; United Kingdom, UK; ZBI, Zarit burden interview scale.
